# Validation of Childhood Rare Epilepsy Social Impact Assessment (CRESIA) to Measure the Social and Family Impact of Rare Childhood Diseases with Epilepsy

**DOI:** 10.3390/jcm11226720

**Published:** 2022-11-13

**Authors:** Rafael Salom, Luis Miguel Aras, Jessica Piñero, Jon Andoni Duñabeitia

**Affiliations:** 1 Centro de Investigación Nebrija en Cognición (CINC), Facultad de Lenguas y Educación, Universidad Nebrija, 28248 Madrid, Spain; 2 Asociación ApoyoDravet, 20009 San Sebastián, Spain; 3 Servicio Navarro de Salud-Osasunbidea, 31010 Navarra, Spain; 4 Fundación Salud Infantil, 03201 Elche, Spain; 5 AcqVA Aurora Center, Department of Languages and Culture, UiT the Arctic University of Norway, 9019 Tromsø, Norway

**Keywords:** rare diseases, epilepsy, social impact, family impact, psychosocial assessment

## Abstract

This study addresses the social relevance of low-prevalence childhood diseases and reports the process of generation and validation of a tool to assess the social impact on the direct family environment and the social context of reference. The aim of the process of construction and validation of this instrument is to provide the field with a tool with the capacity to shed light on the social consequences of suffering from a low-prevalence disease, specifically those comorbid with treatment-resistant epileptic seizures of childhood origin. The instrument here presented and called CRESIA (acronym derived from Childhood Rare Epilepsy Social Impact Assessment) provides valuable information on six specific areas framing health, economic, psychological, social, and child-related stressors, as well as family. CRESIA represents a valid and reliable instrument for family members or primary caregivers of children and adolescents with childhood rare epilepsy.

## 1. Introduction

The present study comprises the creation and validation process of a tool aimed to provide a clearer picture of the specific problems of rare diseases (RDs), specifically childhood rare epilepsies (CREs). RDs are those pathologies that affect a small number of individuals within the general population. The consensus is that a disease is considered rare when its prevalence is less than 5/10,000 persons [1,2,3]. There are currently over 7000 types of RDs, and above 1000 are related to the central nervous system (CNS). More than 50% of CNS-related RDs occur in children and are characterized by being chronic, progressive, and life-threatening [4,5].

Despite symptomatology may vary from one patient to another, a common symptom of CNS-related RDs is the appearance of episodes of epileptic seizures, especially in children (CREs) [5,6]. Epileptic seizures are chronic episodes of the central nervous system that occur unexpectedly and spontaneously. They are triggered by the excessive electrical activity of a group of hyperexcitable neurons and may cause shaking and a change in sensation, leaving the individual feeling confused or dazed. The consequences of epileptic seizures can include cognitive, physical, and social impairment, increasing the risk of mortality in those affected by CNS-related RDs [6,7,8,9].

In the different CREs, epileptic and developmental encephalopathies (including syndromes or diseases in which the etiology and severity of seizures or electrical activity are abnormal) are mainly responsible for severe neurological, cognitive, and behavioral effects. They are also associated with high mortality rates compared with other epilepsies, due to the succession of seizures and genetic abnormalities [10]. In particular, refractory epileptic seizures (resistant to antiepileptic drugs) require special interest as their onset is usually early in life, with seizures that are often intractable and that involve high neurodevelopmental impact, resulting in high socio-economic cost and a marked impact on parental quality life [11,12,13].

Moreover, there is a lack of information and medical advice on the symptoms of CREs, typically because of the lack of specific training of healthcare personnel, which delays diagnosis and treatment [6,14,15,16,17,18,19]. Due to the wide variety of symptoms, there is no unified treatment for each of them, but instead, a plethora of rather complex treatments only aimed at palliating the symptoms are generally proposed [4,18,20,21,22]. Although different types of treatments have been proposed, the vast majority of them rely on pharmacological interventions. According to their year of marketing, these prescription drugs are classified accordingly as first-, second-, or third-generation drugs, and there are currently more than 25 classified drugs approved by the relevant agencies. While pharmacological approaches are widely extended, other invasive therapeutic avenues are used with varying degrees of prevalence, such as surgery or neurostimulation, aimed at controlling the anticonvulsant effects by modulating the neuronal homeostasis of the firing rate [23,24]. Importantly, for the purposes of the current study, not only invasive surgical approaches may result in unforeseen consequences, but antiepileptic pharmacological drugs have also been proven to yield adverse cognitive effects that must be considered and weighed against their potential benefits, as they can have a major impact on both the individual and the family [25]. That, along with the high variability of the symptomatology (e.g., seizures, fever, episodes of absences, or loss of consciousness) and individual differences in the spill-over effects (behavioral disorders, cognitive problems, and sleep disorders), leaves parents with no guidelines for coping with the disease [14,15,16,17]. Hence, it comes as no surprise that the lack of resources and the process of family adaptation to the children’s and adolescent’s circumstances negatively impact the whole family’s health and well-being [14,15,26].

CREs have a wide range of effects on families depending on different factors, such as the severity and complexity of the disease, the social support and the available resources, and the coping skills of the parents [27]. In primary care, some of the most common complaints among the parents of children and adolescents with CREs are fatigue, symptoms of anxiety and depression, high levels of stress, and low levels of self-esteem [15,28]. In addition, they experience a high degree of worry due to repeatedly occurring seizures, which cause harm and endanger the offspring’s survival. All of this leads to a decrease in work productivity, social relationships, and parental self-care [12,29]. One of the factors that significantly increase the emotional burden is disruptive behavior and behavioral disorders, including aggression, self-harm, and tantrums, of their children and adolescents [10,30]. At social events, the family may be embarrassed by such disruptive behavior of the minor, and this leads to further social stigma, due to the social misunderstanding of the complexity of the illness, and parental isolation to avoid awkward moments [27,28,29,31,32].

Research is increasingly focusing on the impact of CREs on the quality of life of families and the affected person. Although it has been highlighted that factors that disrupt parents’ daily lives and their emotional regulation are equally important for the healthy development of the family and their offspring [33], there is still a lack of attention and research on systemic family health interventions [34,35]. Thus, given the high impact of CREs on families, evidence-based solution proposals are needed. As a first necessary step, the identification of the core components of families’ quality of life is essential to be able to carry out appropriate personalized family interventions [14,28,29,32,33].

This study reports on the process of developing and validating an instrument to measure the impact of CREs on families. To date and to the best of our knowledge, there is no specific instrument for analyzing and measuring the social impact of CREs. The instrument here presented, called Childhood Rare Epilepsy Social Impact Assessment (CRESIA), was created to allow new policies and interventions to be generated based on scientific evidence. The next sections present the design and construction process of the instrument, as well as the statistical processes to assess its validity and reliability.

## 2. Materials and Methods

Given the absence of an instrument to collect information that covers the social elements that impact the lives of these families, the general objective of this study was to construct the CRESIA instrument and test its validity and reliability in a Spanish sample. The specific objectives were: (1) to establish the items and questions of the instrument for its Spanish version; (2) to validate the content based on the evaluation of expert judges; (3) to reduce the dimensions of the instrument into factors; and (4) to analyze the reliability and internal consistency of the instrument.

### 2.1. Participants

In the validation of this instrument, a total of 25 expert judges with a background in healthcare (i.e., physiotherapists, social workers, physiotherapists, speech therapists, and occupational therapists) participated. This sample size was considered satisfactory for studies of this nature since the literature recommends using a number close to 10 [36,37,38,39]. The expert judges were selected based on criteria related to their professional experience [40,41].

For the factor analysis and reliability purposes, the chosen sample consisted of 45 Spanish adults (75.6% females) with a mean age of 42.8 years (SD = 6.4) that were parents or legal guardians of children or adolescents that had been diagnosed with RDs comorbid with epilepsy (see Table A1 in the Appendix A) (57.8% females, mean age = 9.9 years, SD = 5.0). Participants who presented clinical symptoms associated with any psychological disorder in the previous 6 months (e.g., major depression or post-traumatic stress disorder) were not allowed to participate in the study. Additionally, information was collected on their professional status and social economic class data. Out of the 45 parents, 36 were employed; 5 were housewives; and 4 were unemployed or inactive. Similarly, out of the 45 parents, 29 belonged to the middle class, 10 to the upper-middle class, 3 to the lower-middle class, and 3 to the lower class. Table 1 contains the data referring to the quantitative socio-demographic questionnaire.

The purpose of the study was explained to the participants before starting, and they signed their informed consent. This study was approved by the Ethics and Research Committee of Universidad Nebrija according to the principles established in the current legislation on clinical research (Organic Law 15/99 of 13 December on the Protection of Personal Data, Law of 41 November regulating Patient Autonomy).

### 2.2. Materials Design and Selection Procedure

For the design and subsequent validation of the Spanish version of the CRESIA instrument, a system of indicators was used in a group of 6 domains of study of social impact: (A) Social; (B) Health; (C) Psychological; (D) Family; (E) Stressors caused by the child; F) Economic. The aim of the process was to construct an instrument capable of measuring this system of indicators and the different domains outlined above. After an exhaustive review of the literature, a bank of a total of 370 items was created that responded to the different domains of the system of indicators. The items were grouped into subcategories (indicators) of the 6 different domains (see Table 2). 

The system of indicators was examined with the collaboration of experts. To identify whether the instrument met the proposed objectives before the judges’ evaluation, the items were first presented to 4 professionals of prestige in the field. Their role was to indicate, with a dichotomous nominal measure (i.e., “good/bad”), whether the items presented were correct or not in terms of their design, grammar, and evaluation strength. An analysis of their agreement was conducted. Jamovi [42] for Windows was used for this and subsequent data analyses.

After this initial process, the screening and selection of the items were carried out. First, a content validity study and selection of the initial version of the instrument were carried out with 25 expert judges with experience in healthcare. The instrument was constructed in such a way that each item could be evaluated by each judge according to its relevance to its indicator. The extent to which each item succeeded in measuring the components associated with the corresponding indicator was assessed. Specifically, a 4-point scale was established: 1 = very little; 2 = little; 3 = quite a lot; 4 = a lot.

Once the instrument was validated, it was necessary to identify the items whose scores had to be reversed, either positively or negatively, to claim that the final score of the instrument was correct. To do this, all the items were given to two professionals, whose role was to decide whether the items had to be inverted or not (so that the final score corresponded to the type of measure we wanted to evaluate). With the data collected, a table of frequencies and inter-rater reliability was drawn up to observe the degree of agreement between the two judges.

Finally, a factor analysis and reliability study of the instrument were carried out after piloting it with the target population in its final development stage. This pilot study consisted of administering the instrument to 45 families with children and adolescents with RDs comorbid with epileptic seizures. The questionnaire used a 1-to-5 Likert-type scale (with 5 being the value that represented the greatest impact). (Bear in mind that some items were reversed in order to be able to interpret them correctly.)

After piloting, a first construct validity analysis was carried out to group the variables into different factor groups and to explore whether the relationships among the variables defined an invariant dimensional structure in the questionnaire. A principal components analysis was used to reduce the dimensionality of the information contained in the indicators. With this, the initial indicators were explored and grouped into domains. Secondly, exploratory factor analyses were carried out for each domain. The aim was to explain the common variance among the variables with the smallest number of indicators and items. In this way, all the items showing mutual relationships were grouped or saturated in the same indicator. To evaluate the output model, Bartlett’s test of sphericity, the Kaiser–Meyer–Olkin measure of sampling adequacy (KMO), and the cumulative variance of each factor was computed.

To analyze the tool’s reliability, a study of Cronbach’s alpha coefficient was carried out. This determined the internal consistency of the instrument, both for each of the indicators and for the domains, as well as for the entire instrument, to refine and consolidate it as a whole.

## 3. Results

The results of the validation process of the Childhood Rare Epilepsy Social Impact Assessment (CRESIA) instrument are described below, showing the data collected to determine the validity and reliability of the instrument.

### 3.1. Validity

The inter-rater agreement level of the four raters showed a very high level of convergence in their responses (agreement of 83%), indicating that the instrument met the proposed objectives of the design, grammar, and strength of assessment of the items that were subsequently presented to the 25 judges.

Next, the average responses of the 25 independent judges on the four-point scale on the appropriateness of each item for each indicator (1 = very little; 2 = little; 3 = quite a lot; 4 = a lot) were analyzed. The mean response to the items was 3.65 out of a total of 4 (range: 3.2–3.96), which shows that the items correctly evaluated the indicators they belonged to. The descriptive statistics of all the items that the judges evaluated and that were used to design the CRESIA instrument were reinforced by an agreement of 91.25% among all the judges (range of % agreement of each item: 80–99).

The degree of agreement between the two evaluators in charge of identifying the elements that required inverting their score to obtain timely results was calculated using Cohen’s kappa. We found 100% agreement between the two evaluators (K = 1.00; *p* < 0.001; Z = 19.2). A total of 137 out of the 370 items required reversing their scoring, and 233 were considered direct items for which the score was left unchanged.

### 3.2. Principal Component Analysis (PCA)

The principal component analysis (PCA) proposed a five-factor (or domain) composition for the CRESIA instrument. The analysis was subjected to an orthogonal rotation (varimax; [43,44,45]). The instrument, after the PCA and its subdivision into five factors, showed a KMO sampling suitability measure of 0.81. Bartlett’s sphericity test was significant (*p* < 0.001), with a cumulative variance of 100%. Factor loadings were greater than the minimum of 0.3 in all elements. The five factors that subdivide the instrument into different evaluation scales or domains explain the total variance of the instrument as follows: factor 1 (Domain D, Family), 21.7% of the variance; factor 2 (Domain E, Stressors caused by the child), 21.5%; factor 3 (Domain B, Health), 21.2%; factor 4 (Domain C, Psychological), 18%; and finally, factor 5 (Domain A, Social) represents the remaining 17.6% of the total variance of the instrument.

### 3.3. Exploratory Factor Analysis (EFA)

After the PCA, which showed that the CRESIA instrument should be composed of five factors, an exploratory factor analysis of all the factors was performed. The variables were subjected to the minimum residual extraction method and an oblique rotation (oblimin; [43,44,45]).

#### 3.3.1. EFA Domain A (Social)

On the scale of Domain A (Social), after the EFA, its subdivision into two factors (indicators) was suggested. A KMO sampling suitability measure of 0.75 was observed. Bartlett’s sphericity test was significant (*p* < 0.001), with a cumulative variance of 53.4%. Factor loadings were greater than the minimum of 0.3 in all elements. Factor 1 (Indicator A.1, Perceived burden) represented 28.7% of the variance, and factor 2 (Indicator A.2, Social support and self-concept) represented 24.7% of the remaining variance.

#### 3.3.2. EFA Domain B (Health)

The scale of Domain B (Health) did not require subdivision, since it could be explained with a single factor (Indicator B.1, General health), as indicated by the exploratory factor analysis carried out in this case. A KMO sampling suitability measure of 0.68 was observed. Bartlett’s sphericity test was significant (*p* < 0.001), with a cumulative variance of 51.2%. Factor loadings were greater than the minimum of 0.3 in all elements.

#### 3.3.3. EFA Domain C (Psychological)

In the scale of Domain C (Psychological), a larger subdivision was required due to a large number of items, and the results of the EFA proposed a subdivision in four factors. For the joint analysis of these factors, a KMO sampling suitability measure of 0.76 was observed. Bartlett’s sphericity test was significant (*p* < 0.001), with a cumulative variance of 71.3%. Factor loadings were greater than the minimum of 0.3 in all elements. Factor 1 (Indicator C.2, Work stress) represented 25.21% of the variance; factor 2 (Indicator C.1, Emotional condition), 22.72%; factor 3 (Indicator C.4, Work self-concept), 15.3%; and finally, factor 4 (Indicator C.3, Self-concept) 8.1% of the remaining variance.

#### 3.3.4. EFA Domain D (Family)

The EFA endorsed a subdivision of the indicators of this domain into three factors. A KMO sampling suitability measure of 0.74 was observed. Bartlett’s sphericity test was significant (*p* < 0.001), with a cumulative variance of 53%. Factor loadings were greater than the minimum of 0.3. in all elements. Factor 1 (Indicator D.1, Perceived family support) represented 25.3% of the variance; factor 2 (Indicator D.2, Family satisfaction) represented 16.8%; and factor 3 (Indicator D.3, Impact on the family environment) corresponded to 10.8% of the remaining variance.

#### 3.3.5. EFA Domain E (Stressors Caused by the Child)

The EFA suggested a subdivision into four factors. A KMO sampling suitability measure of 0.58 was observed. Bartlett’s sphericity test was significant (*p* < 0.001), with a cumulative variance of 60.2%. Factor loadings were greater than the minimum of 0.3. in all elements. Factor 1 (Indicator E.1, Social manifestations of the child) represented 18.4% of the variance; factor 2 (Indicator E.3, Emotional manifestations of the child) represented 17.1%; factor 3 (Indicator E.2, Behavioral manifestations of the child) represented 12.7%; and factor 4 (Indicator E.4, Physiological and biological manifestations of the child) represented 12% of the remaining variance.

### 3.4. Reliability

The global reliability index of the CRESIA tool was very high. CRESIA had a Cronbach’s Alpha coefficient of 0.98, showing excellent internal consistency. Regarding the reliability index within each of the different domains, equally good internal consistency was found as follows: Domain A, Social, α = 0.91; Domain B, Health, α = 0.95; Domain C, Psychological, α = 0.98; Domain D, Family, α = 0.89; Domain E, Stressors caused by the child, α = 0.82.

## 4. Discussion

In view of the high prevalence of childhood rare epilepsy in rare diseases [4,5] and considering their great impact in multiple areas that affect the quality of life not only of the children but also of their caregivers [14,15,26], this study sought to develop and validate a measurement instrument to characterize and quantify their social impact on families with children and adolescents. Given the lack of materials to adequately quantify and qualify this impact and the urge to provide an adjusted response and being the first of this nature in the field, the instrument here presented stands as a benchmark tool for families with offspring with CREs, opening doors to evidence-based intervention plans at social, political, and biomedical levels.

The validity of Childhood Rare Epilepsy Social Impact Assessment (CRESIA) was confirmed by a group of 25 expert judges that were selected based on criteria related to their professional experience [40]. Such an expert’s sample size is sufficiently large to grant strength to the validation process, according to studies exploring the best way to determine the content validity of an instrument of this nature [36,37,38,39]. Importantly, the scores collected for each of the items indicated that the overall evaluation completed by the judges was very positive and that the instrument could be safely considered suitable for measuring the social impact on families with minors with CREs. Additionally, the content validity of the instrument was endorsed by the 80% agreement among the experts on the relevance of each item for measuring what it was meant to measure. As suggested by Voutilainen and Liukkonen [46], if 80% of the experts agree that an item is valid, it can be incorporated into the instrument with sufficient confidence. The high values obtained across experts reinforced the idea of a correct instrument design that is adequate to analyze the intended construct as a whole. Further analyses based on a PCA and an EFA also confirmed that the five-factor model (excluding the Economic factor that, given its different measuring scales, did not allow for a joint analysis) and its sub-scales correctly fitted the data, again endorsing the high construct validity of the instrument. This result aligned with the original expectations that drove the creation of the tool and demonstrated that the five dimensions of interest should be treated independently: Social, Health, Psychological, Family, and Stressors caused by the child. Hence, the high level of internal consistency of the entire scale indicated that the calculation of the total score on the basis of the different indicators that map into the different domains is appropriate and useful for research and/or clinical screening activities.

This instrument was also piloted in a real representative sample, which allowed us to determine its reliability. During the development process of the tool, the active participation of parents of individuals affected by CREs was taken into account in order to ensure that the needs of the target families were met, working together with them to shape and guide the work as it developed. The analysis of the data obtained in this collaborative process led to conclude that both globally and in each of the target domains, the instrument presented a high degree of internal consistency and it is assumed that the items measure the same construct. Hence, in light of these findings, it could be confidently assumed that the decisions derived from the interpretation of the scores obtained with this instrument could significantly impact the person and their context by allowing tailored and focused interventions to be conducted [47]. Therefore, it is concluded that CRESIA is a reliable instrument with high internal consistency. However, the data used were only cross-sectional, leaving the future goal of assessing the sensitivity of the scale to changes over time.

All in all, this instrument is proposed as an effective tool to analyze and describe the social impact in future case and group studies on specific CRE samples. The areas that can be assessed with this tool give way to the quantification and characterization of social problems previously discussed by different authors in the context of rare diseases, specifically targeting the impacts on the social, psychological, family, health, and economic aspects of this population [12,14,33,34,35]. Thus, to the best of our knowledge, this study provides the first valid and reliable instrument to assess the needs of patients with CREs and their families, making it possible to identify those aspects that disrupt their quality of life as a result of the rare disease. Additionally, in our view, this tool provides clinicians with an instrument with the capacity to help health professionals to monitor the potentially masked or unforeseen psychosocial impact of a given surgical or pharmacological treatment. Thus, over and above exploring the social consequences of medical actions, this instrument could be used to develop science-based clinical and social policies specifically tailored to the social context of each patient, favoring multidisciplinary integrative approaches.

Admittedly, this study is the first in a long-range plan of research, and additional work is clearly needed to expand the use of this tool across rare diseases in different cultures and with different languages. Furthermore, this tool has the potential to be expanded to other rare diseases, and future work should focus on targeting different rare diseases to better characterize their reality at an integrative level. With this in mind, we are currently working on a revised version that focuses on Dravet Syndrome, where the origin of the disease, gene mutation, or the phenotypes affected in each case are specifically explored. Moreover, our team is currently creating a normative database with respondents from families with healthy minors, in an attempt to elaborate a set of reference comparison norms that could allow us to establish the thresholds or cut-off points of what could be considered a family at risk or in a deprived situation. We sincerely hope that other researchers will find in this tool and this approach an opportunity to develop adaptations to cover relevant aspects of other rare diseases. Nonetheless, being the first of its kind, this study already represents an opportunity to evaluate and improve therapeutic and social interventions specifically targeting families with offspring with CREs. It comprises a broad assessment of the social impact of CREs, which is of utmost importance for inclusive societies aiming at offering a viable solution to the specific needs of families with limited access to resources and information. We firmly believe that the use of Childhood Rare Epilepsy Social Impact Assessment (CRESIA) and the development of similar tools will improve and guide the implementation of policies for social inclusion and attention to diversity.

## Figures and Tables

**Table 1:** Socio-demographic data.

	Average	Median	SD	Min	Max
Participant’s age (in years)	42.78	43	6.37	31	56
No. of inhabitants in the household	3.69	4	0.67	2	5
Offspring’s age (in years)	9.9	10	5	1	17
Age at which the offspring was diagnosed (in years)	3.11	2	3.6	0	16
Time elapsed between first symptoms and diagnosis (in months)	27.1	11	32	0	100
No. of epileptic seizures in a week	14.1	4	13.8	1	31

**Table 2:** Structure of CRESIA.

Domain	Indicator	No. of Items
A. Social	A.1. Perceived burden	22
	A.2. Social support and self-concept	21
B. Health	B.1. General health	36
C. Psychological	C.1. Emotional condition	146
	C.2. Work stress	25
	C.3. Self-concept	23
	C.4. Work self-concept	16
D. Family	D.1. Perceived family support	6
	D.2. Family satisfaction	6
	D.3. Impact on the family environment	5
E. Stressors caused by the child	E.1. Social manifestations of the child	5
	E.2. Behavioral manifestations of the child	5
	E.3. Emotional manifestations of the child	5
	E.4. Physiological and biological manifestations of the child	4
F. Economic	F.1.1. Monthly income	4
	F.1.2. Direct costs	31
	F.1.3. Indirect costs	10

## Data Availability

All the data obtained in this study are available upon request to R.S. (rsalom@nebrija.es).

## Appendix A

**Table A1:** Diagnostic of offspring disease.

Diagnosis	Frequency
Dravet syndrome	N = 17 (37.8%)
CDKL5 deficiency syndrome	N = 7 (15.55%)
SCN8A mutation	N = 6 (13.3%)
PCDH19 mutation	N = 3 (6.7%)
CACNA1A mutation	N = 3 (6.7%)
Lennox–Gastaut syndrome	N = 2 (4.4%)
Other epilepsies	N = 7 (15.55%)

## References

[1] Nguengang Wakap S., Lambert D.M., Olry A., Rodwell C., Gueydan C., Lanneau V., Murphy D., Le Cam Y., Rath A. (2020). Estimating cumulative point prevalence of rare diseases: Analysis of the Orphanet database. Eur. J. Hum. Genet..

[2] Orphanet, Procedural document on the Orphanet nomenclature and classification of rare diseases, Orphanet 2020, Number 2. https://www.orpha.net/orphacom/cahiers/docs/GB/eproc_disease_inventory_R1_Nom_Dis_EP_04.pdf.

[3] Van Der Zeijden A., Huizer J. (2010). Recommendations for the development of national plans for rare diseases. Orphanet J. Rare Dis..

[4] Schieppati A., Henter J.-I., Daina E., Aperia A. (2008). Why rare diseases are an important medical and social issue. Lancet.

[5] Williams J., Nakas N., Huml R.A. (2021). Central Nervous System Rare Disease Drug Development. Rare Disease Drug Development.

[6] Fisher R.S., Acevedo C., Arzimanoglou A., Bogacz A., Cross J.H., Elger C.E., Engel J., Forsgren L., French J.A., Glynn M. (2014). ILAE Official Report: A practical clinical definition of epilepsy. Epilepsia.

[7] Fisher R.S., Cross J.H., French J.A., Higurashi N., Hirsch E., Jansen F.E., Lagae L., Moshé S.L., Peltola J., Perez E.R. (2017). Operational classification of seizure types by the International League Against Epilepsy: Position Paper of the ILAE Commission for Classification and Terminology. Epilepsia.

[8] World Health Organization (2019). Epilepsy: A Public Health Imperative: Summary.

[9] World Health Organization (2019). A report about epilepsy, World Health Organization. https://www.who.int/es/news-room/fact-sheets/detail/epilepsy.

[10] Feizi A., Najmi B., Salesi A., Chorami M., Hoveidafar R. (2014). Parenting stress among mothers of children with different physical, mental, and psychological problems. J. Res. Med. Sci..

[11] Farrace D., Tommasi M., Casadio C., Verrotti A. (2013). Parenting stress evaluation and behavioral syndromes in a group of pediatric patients with epilepsy. Epilepsy Behav..

[12] Wirrell E.C., Wood L., Hamiwka L.D., Sherman E.M. (2008). Parenting stress in mothers of children with intractable epilepsy. Epilepsy Behav..

[13] Dawes A., Attipoe S., Mittlesteadt J., Glynn P., Rust S., Debs A., Patel A.D. (2020). Measuring the impact of epilepsy on families. Epilepsy Behav..

[14] Beghi E. (2016). Addressing the burden of epilepsy: Many unmet needs. Pharmacol. Res..

[15] Dellve L., Samuelsson L., Tallborn A., Fasth A., Hallberg L.R.-M. (2006). Stress and well-being among parents of children with rare diseases: A prospective intervention study. J. Adv. Nurs..

[16] Grut L., Kvam M.H. (2013). Facing ignorance: People with rare disorders and their experiences with public health and welfare services. Scand. J. Disabil. Res..

[17] Huyard C. (2009). What, if anything, is specific about having a rare disorder? Patients’ judgements on being ill and being rare. Heal. Expect..

[18] Mahendran M., Speechley K.N., Widjaja E. (2017). Systematic review of unmet healthcare needs in patients with epilepsy. Epilepsy Behav..

[19] Wu K.N., Lieber E., Siddarth P., Smith K., Sankar R., Caplan R. (2008). Dealing with epilepsy: Parents speak up. Epilepsy Behav..

[20] Dodge J.A., Chigladze T., Donadieu J., Grossman Z., Ramos F., Serlicorni A., Siderius L., Stefanidis C.J., Tasic V., Valiulis A. (2010). The importance of rare diseases: From the gene to society. Arch. Dis. Child..

[21] Kwint H.F., Faber A., Gussekloo J., Bouvy M. (2012). The contribution of patient interviews to the identification of drug-related problems in home medication review. J. Clin. Pharm. Ther..

[22] Zurynski Y., Frith K., Leonard H., Elliott E. (2008). Rare childhood diseases: How should we respond?. Arch. Dis. Child..

[23] Liu X., Carney P.R., Bussing R., Segal R., Cottler L.B., Winterstein A.G. (2017). Trends in Antiepileptic Drug Use in Children and Adolescents With Epilepsy. Pediatr. Neurol..

[24] Mulroe F., Lin W.-H., Scott C.M.-G., Aourz N., Fan Y.N., Coutts G., Parrish R.R., Smolders I., Trevelyan A., Wykes R.C. (2022). Targeting firing rate neuronal homeostasis can prevent seizures. Dis. Model. Mech..

[25] Braun K.P. (2017). Preventing cognitive impairment in children with epilepsy. Curr. Opin. Neurol..

[26] Neely-Barnes S.L., Dia D.A. (2008). Families of children with disabilities: A review of literature and recommendations for interventions. J. Early Intensiv. Behav. Interv..

[27] Camfield C., Camfield P. (2008). Twenty years after childhood-onset symptomatic generalized epilepsy the social outcome is usually dependency or death: A population-based study. Dev. Med. Child Neurol..

[28] Camfield C., Breau L., Camfield P. (2008). Impact of Pediatric Epilepsy on the Family: A New Scale for Clinical and Research Use. Epilepsia.

[29] Mu P.-F. (2008). Transition experience of parents caring of children with epilepsy: A phenomenological study. Int. J. Nurs. Stud..

[30] Vlaskamp D.R., Shaw B.J., Burgess R., Mei D., Montomoli M., Xie H., Myers C.T., Bennett M.F., XiangWei W., Williams D. (2018). *SYNGAP1* encephalopathy. Neurology.

[31] Austin J.K., McDermott N. (1988). Parental Attitude and Coping Behaviors in Families of Children with Epilepsy. J. Neurosci. Nurs..

[32] Chong L., Jamieson N.J., Gill D., Singh-Grewal D., Craig J.C., Ju A., Hanson C.S., Tong A. (2016). Children’s Experiences of Epilepsy: A Systematic Review of Qualitative Studies. Pediatrics.

[33] Rodenburg R., Stams G.J., Meijer A.M., Aldenkamp A.P., Deković M. (2005). Psychopathology in Children with Epilepsy: A Meta-Analysis. J. Pediatr. Psychol..

[34] Dekovic M., Janssens J.M.A.M., As N.M.C. (2003). Family Predictors of Antisocial Behavior in Adolescence. Fam. Process.

[35] Rodenburg R., Meijer A.M., Deković M., Aldenkamp A.P. (2006). Family Predictors of Psychopathology in Children with Epilepsy. Epilepsia.

[36] Mayaute L.M.E. (1969). Cuantificación de la validez de contenido por criterio de jueces. Rev. Piscol..

[37] Gable R.K., Wolf M.B. (2012). Instrument Development in the Affective Ambit: Measuring Attitudes and Values in Corporate and School Settings: 36 (2nd 1993 ed.).

[38] Hyrkäs K., Appelqvist-Schmidlechner K., Oksa L. (2003). Validating an instrument for clinical supervision using an expert panel. Int. J. Nurs. Stud..

[39] Lynn M.R. (1986). Determination and Quantification Of Content Validity. Nurs. Res..

[40] Rubio D.M., Berg-Weger M., Tebb S.S., Lee E.S., Rauch S. (2003). Objectifying content validity: Conducting a content validity study in social work research. Soc. Work Res..

[41] Sampieri R.H., Collado C.F., Lucio P.B., Valencia S.M., Torres C.P.M. (2014). Metodología de la Investigación.

[42] (2022). The jamovi project. jamovi. https://www.jamovi.org.

[43] Beavers A.S., Lounsbury J.W., Richards J.K., Huck S.W., Skolits G.J., Esquivel S.L. (2013). Practical considerations for using exploratory factor analysis in educational research. Pract. Assess. Res. Eval..

[44] Conway J.M., Huffcutt A.I. (2003). A Review and Evaluation of Exploratory Factor Analysis Practices in Organizational Research. Organ. Res. Methods.

[45] Costello A.B., Osborne J.W. (2005). Best practices in exploratory factor analysis: Four recommendations for getting the most from your analysis. Pract. Assess. Res. Eval..

[46] Voutilainen P., Liukkonen A. (1995). Senior Monitor—laadun arviointimittarin sisällön validiteetin määrittäminen. Hoitotiede.

[47] Prieto G., Delgado A.R. (2010). Fiabilidad y validez. Pap. Psicol..

